# Boosting T cell immunity against cytomegalovirus: a potential strategy combating human aging and age-related diseases

**DOI:** 10.1038/s41392-023-01590-6

**Published:** 2023-09-01

**Authors:** Matthias Wirth, Walburgis Brenner, Oliver H. Krämer

**Affiliations:** 1 Department of Hematology, Oncology and Cancer Immunology, Campus Benjamin Franklin, Charité - Universitätsmedizin Berlin, corporate member of Freie Universität Berlin and Humboldt-Universität zu Berlin, Berlin, Germany; 2 University Medical Center Göttingen, Department of General, Visceral and Pediatric Surgery, Göttingen, Germany; 3 German Cancer Consortium (DKTK), German Cancer Research Center (DKFZ), Heidelberg, Germany; 4 Clinic for Obstetrics and Women’s Health, University Medical Center, Johannes Gutenberg University Mainz, Langenbeckstr. 1, 55131 Mainz, Germany; 5 Department of Toxicology, University Medical Center, Mainz, Germany

**Keywords:** Senescence, Molecular medicine

In a recent publication in *Cell*, Hasegawa and colleagues reveal that cytotoxic CD4-positive cytotoxic T lymphocytes (CD4^+^ CTLs) eliminate senescent fibroblasts with a surface expression of the immunologically important human leukocyte antigen class II (HLA-II) and the human cytomegalovirus glycoprotein B (HCMV-gB).^1^ This implies that a virome-immune cell interaction controls the fate of human skin cells that are linked to the aging process and associated diseases.

A previously unknown rise in life expectancy of current human societies strengthens the desire to uncouple the aging process from age-associated risk factors and chronic disorders. The persistence of old and damaged cells in tissues hampers this goal. Cells with such a senescent phenotype carry typical morphological and biochemical alterations. For example, cyclin-dependent kinases induce cell cycle progression for cell growth and renewal. Cyclin-dependent kinase inhibitors (e.g., p16^INK4A^ and p21^CIP1^) attenuate these processes and are biochemical markers of senescent cells. Notably, senescent cells are not inert but contribute to pathological functions in inflammation, cancer, age-related diseases, and other ailments.

Hasegawa and colleagues collected normal truncal skin samples that were removed surgically from females. The authors categorized them into two groups that they termed young (16–28 years) and old skin (53–74 years). Fibroblasts are the main cell type of our connective tissues. As anticipated, younger skin tissues (*n* = 23) compared to older skin tissues (*n* = 31) had less p16^INK4A^-positive and p21^CIP1^-positive senescent fibroblasts (Fig. 1). Curiously, this rise in aged fibroblasts did not significantly upsurge over lifetime in the samples collected from females in the ages from 53–74 years. This prompted the authors to investigate why the gradual accumulation of senescent fibroblasts reached a plateau. The analysis of skin-resident blood cells disclosed significantly higher amounts of CD4^+^ and CD8^+^ CTLs in older than in younger skin samples, although the total levels of Th1 lymphocytes favoring cell-based immune responses was overtly lower in older skin. Notably, the levels of CD4^+^ CTLs inversely correlated with the levels of p16^INK4A^-positive fibroblasts in old dermis. The chemokine CXCL9 recruits CD4^+^ CTLs into skin and was upregulated in old versus new skin (Fig. 1).

**Fig. 1:**
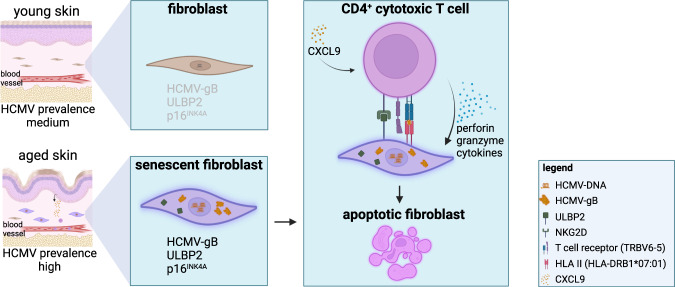
Cellular senescence is characterized by a replicative arrest that can be induced by various stress stimuli. In aged skin, the population of senescent cells can be controlled by CXCL9-attracted cytotoxic CD4^+^ T lymphocytes (CD4^+^ CTLs). HLA-II-dependent presentation of human cytomegalovirus glycoprotein B (HCMV-gB) and high expression of the activating surface receptor of cytotoxic immune cells (NKG2D) and its HLA-I related ligand UL16-binding protein-2 (ULBP2) mark senescent fibroblasts for CD4^+^ CTLs-mediated killing. Figure created with BioRender.com

Human cytomegalovirus (HCMV, human β-herpes virus 5) occurs with a prevalence between 60-100% worldwide.^2^ Adult immunocompetent hosts usually control lifelong, latent HCMV infections without symptoms. HCMV has a viral envelope with a lipid double membrane that is studded with various glycoproteins, such as HCMV-gB, HCMV-gH, HCMV-gL, and HCMV-gM.^2^ Hasegawa et al. show that HCMV DNA and RNA expression and, in particular, high HCMV-gB levels are predominantly expressed in senescent fibroblasts and that this increases with age.^1^ Specific immune responses exerted by HLA-I-restricted CD8^+^ CTLs but also HLA-II-restricted CD4^+^ lymphocytes, control HCMV infection early and during the development of immunity. Notably, HCMV suppresses rather HLA-I than HLA-II, indicating long-term virus control through CD4^+^ CTLs.^2^

There is an intriguing, beneficial interaction between HCMV and CD4^+^ CTLs (Fig. 1). T Cell Receptor-β Variable 6–5 (TRBV6-5) is a part of the variable domain of the T cell receptor (TCR), which permits antigen recognition. TRBV6-5 specifically recognizes the HCMV-gB epitope DYSNTHSTRYV when it is presented on the HLA-II antigen HLA-DRB1*07:01 on senescent fibroblasts. Sequencing of the TCR revealed the existence of TRBV6-5 positive T cell clones in old skin tissue. To identify T cell-mediated killing of senescent fibroblasts, Hasegawa and colleagues performed autologous co-culturing of T cells and fibroblasts. The bulk of senescent fibroblasts that went into cell death were HMCV-gB positive. Pre-incubation with an HLA-DR-specific antibody blocked CD4^+^ CTL-mediated cell death, confirming death via HLA-II signaling. The authors demonstrated specificity for HCMV-gB by showing that HCMV-gH failed to activate CD4^+^ CTLs. Apparently, expression of HCMV-gB in the absence of lytic infection, due to incomplete replication in senescent fibroblasts, is a protective mechanism that prevents further spread of HCMV. High levels of HLA-II on senescent fibroblasts allowed their elimination by CD4^+^ CTLs, demonstrating a new level of immunosurveillance of aged cells.^1^

The work by Hasegawa and colleagues poses provocative questions. Why has an interplay between HCMV with senescent and immune cells evolved? A possible explanation is that a longer vitality of HCMV-infected hosts prolongs the time in which they can pass on HCMV if it is reactivated. Likewise, is the immunological control of virus-infected cells the key to reduce skin aging? Neither for safety nor for ethical reasons, a deliberate infection of humans with replication-competent viruses can be justified. The last years of the COVID-19 crisis, the AIDS crisis, and the Spanish flu are dismal examples of how uncontrolled viral spread has caused socioeconomic and personal burdens.

The very high prevalence of HCMV in humans may still offers an avenue to target infected senescent cells—through boosting CD4^+^/CD8^+^ CTL—and natural killer cell-driven immune responses. How might this be achieved? HCMV replicates its double-stranded DNA in the host cell nucleus. HCMV replication could e.g., be augmented by a drug-induced hyperacetylation of lysine residues within proteins. This is possible with the well-established anti-epileptic, mood-stabilizing histone deacetylase inhibitor (HDACi) valproic acid.^3^ HDACi have been intensively tested in clinics and some have been approved for the treatment of blood cancers. These drugs evolved from evolutionary old endogenous and microbial compounds (e.g., the fatty acid butyrate).^4^ Whether the intake of such agents can modulate interactions of infected host cells and CD4^+^ CTL and if this exceeds disadvantageous side-effects is unknown. The still open mechanisms through which HCMV-gB levels rise in senescent cells may likewise provide opportunities to stall aging processes.

Hasegawa and colleagues analyzed only female skin samples. A missing sex tropism of HCMV makes it very likely that male skin behaves similarly. As skin samples were surgery leftover material, the donors’ health states may have impacted the number of senescent cells. It additionally needs consideration that the older skin group was from individuals aged over 50 years, suggesting menopausal life. Estrogen and testosterone critically impact human aging processes.^5^ Hormonal changes—alone or in conjunction with HCMV and immune cells—might have contributed to the significant delay in a further accumulation of senescent cells in this age group.

In sum, Hasegawa and colleagues provide human data supporting a new framework for the interactions between viruses and their hosts’ immune systems.

## References

[1] Hasegawa T (2023). Cytotoxic CD4(+) T cells eliminate senescent cells by targeting cytomegalovirus antigen. Cell.

[2] Manandhar T, Ho GT, Pump WC, Blasczyk R, Bade-Doeding C (2019). Battle between host immune cellular responses and HCMV immune evasion. Int. J. Mol. Sci..

[3] Michaelis M (2004). Increased human cytomegalovirus replication in fibroblasts after treatment with therapeutical plasma concentrations of valproic acid. Biochem. Pharmacol..

[4] Mustafa AM, Krämer OH (2023). Pharmacological modulation of the crosstalk between aberrant Janus kinase signaling and epigenetic modifiers of the histone deacetylase family to treat cancer. Pharmacol. Rev..

[5] Majidian M, Kolli H, Moy RL (2021). Management of skin thinning and aging: review of therapies for neocollagenesis; hormones and energy devices. Int. J. Dermatol..

